# High Performance Amplifier Element Realization via MoS_2_/GaTe Heterostructures

**DOI:** 10.1002/advs.201700830

**Published:** 2018-01-15

**Authors:** Xiao Yan, David Wei Zhang, Chunsen Liu, Wenzhong Bao, Shuiyuan Wang, Shijin Ding, Gengfeng Zheng, Peng Zhou

**Affiliations:** ^1^ State Key Laboratory of ASIC and System School of Microelectronics Fudan University Shanghai 200433 China; ^2^ Laboratory of Advanced Materials Department of Chemistry Collaborative Innovation Center of Chemistry for Energy Materials Fudan University Shanghai 200433 China

**Keywords:** 2D materials, bipolar junction transistor, current amplification, van der Waals heterostructure

## Abstract

2D layered materials (2DLMs), together with their heterostructures, have been attracting tremendous research interest in recent years because of their unique physical and electrical properties. A variety of circuit elements have been made using mechanically exfoliated 2DLMs recently, including hard drives, detectors, sensors, and complementary metal oxide semiconductor field‐effect transistors. However, 2DLM‐based amplifier circuit elements are rarely studied. Here, the integration of 2DLMs with 3D bulk materials to fabricate vertical junction transistors with current amplification based on a MoS_2_/GaTe heterostructure is reported. Vertical junction transistors exhibit the typical current amplification characteristics of conventional bulk bipolar junction transistors while having good current transmission coefficients (α ∼ 0.95) and current gain coefficient (β ∼ 7) at room temperature. The devices provide new attractive prospects in the investigation of 2DLM‐based integrated circuits based on amplifier circuits.

Since the basic concept of the bipolar junction transistor (BJT) was patented by Shockley in 1948 and experimentally realized in 1951,^1^ BJTs have launched the microelectronics revolution that led to the Information Age. Until the widespread emergence of complementary metal oxide semiconductor (CMOS) technology in the 1980s, the BJT was the dominant semiconductor technology in microelectronic manufacturing. Compared to CMOSs, BJTs exhibit a higher output current and larger transconductance per unit length, faster switching speeds (particularly under capacitive loading), and excellent properties for many analog and amplifier applications.^2^, ^3^ Thus, this creative three‐terminal (emitter–base collector) device, which acts like a transistor, can be used to build a wide variety of electronic circuits. Modern applications of BJTs have changed; the most notable include high‐speed digital integrated circuits in mainframe computers, precision analog circuits, and amplifier circuits found in radio communications systems. Hence, BJTs still occupy a significant portion of the global semiconductor market. Although the primary drawbacks of BJT circuits (compared to CMOS) include larger direct current (DC) power dissipation and fabrication complexity, BJTs remain the first choice to meet high‐frequency requirements and fast switching speeds. BJTs generally consist of two back‐to‐back pn junctions (p–n–p or n–p–n, depending on the doping polarity). To achieve its high‐frequency and high‐amplification characteristics, the intermediate n or p region should be infinitesimally thin. However, the ultrathin base region is difficult to produce using the conventional processing methods because conventional bulk semiconductors (e.g., Si, Ge, and III–IV materials) limit the current amplification. Therefore, we adopted 2D materials as the base region, which can be as thin as an atom.

The discovery of graphene, one of the most famous 2D layered materials (2DLMs), has been a strong boost for world‐wide research of 2DLMs with diverse electronic properties over the past decade.^4^, ^5^, ^6^, ^7^, ^8^, ^9^, ^10^, ^11^, ^12^, ^13^ Because the thickness of a 2DLM monolayer can be as thin as an atom and the surface is free of dangling bonds, 2DLMs surpass typical nanostructures that are plagued by dangling bonds and trap states. Neighboring 2DLM layers usually interact with each other by van der Waals force,^14^, ^15^ which allows for the integration of highly disparate materials with crystal lattice mismatching. There is considerable freedom in integrating 2DLMs and various nanoscale materials to create a set of diverse van der Waals heterostructures (vdWHs), with functions that could not be achieved previously. Until now, in addition to basic circuit elements such as transistors and CMOSs^16^, ^17^, ^18^, ^19^ based on mechanically exfoliated 2DLMs, more complex circuits have also been achieved recently that were based on vdWHs,^20^, ^21^, ^22^, ^23^, ^24^ such as memories, detectors, and sensors. Nevertheless, as an important part of integrated circuits, 2DLM‐based amplifier circuits have rarely been studied.

Considering the difficulty doping in 2DLMs and the small current of 2DLMs devices, the integration of 3D materials and 2DLMs with different polarity can overcome most challenges. In this work, we report the preparation and characterization of vertical bipolar junction transistors assembled using a GaTe/MoS_2_ heterostructure. MoS_2_ is an ideal material for the base because of its excellent electrical performance.^25^, ^26^, ^27^, ^28^, ^29^ Because the requirement for the emitter region is to provide a large number of holes to inject into the base region in the amplification process, the highly p‐doped GaTe is ideal for the emitter region. Thus, the GaTe–MoS_2_ interface acts as a base–emitter junction. The slightly p‐doped Si acts as the collector, and the MoS_2_–Si interface acts as the base–collector junction. The doping for different regions of this vertical heterostructure bipolar junction transistor (HBT) is more precise than electrostatic doping with multigate control,^30^ which requires at least six voltages. Thus, electrostatic doping with multigate control is not good for practical applications or for the fabrication of integrated circuits due to its buried gates. Instead, the vertical HBT exhibits typical characteristic curves (i.e., conventional post‐Si BJT), and the current gain coefficient β can reach 7 at room temperature.

Vertical bipolar junction transistors were fabricated by transferring mechanically exfoliated GaTe and MoS_2_ thin films sequentially onto a slightly p‐doped Si substrate to form a GaTe/MoS_2_/Si sandwich structure. The fabrication process flow diagram is shown in **Figure** **1**. The optical measurement of the device is shown in **Figure** **2**a, with the circled regions (red and blue) representing MoS_2_ and GaTe flakes, respectively. Both of the MoS_2_ and GaTe flakes are layered materials stacked by van der Waals force; thus, few‐layer materials can be peeled off from their bulk states, along the van der Waals gaps. Figure 2b shows a cross‐section schematic diagram of the device, the device structure, and the formation of two van der Waals heterostructures. Figure 2c shows the typical photoluminescence (PL) spectra for the GaTe–MoS_2_ overlapped area, individual MoS_2_, and individual GaTe under 532 nm laser excitation. Under strong laser intensity, individual MoS_2_ show mild PL signals of both the A‐excitonic peak at 1.81 eV and B‐excitonic peak at 1.98 eV. We note that the 1.4 eV peak associated with the indirect bandgap of MoS_2_ could not be observed. There might be three possible origins: the hot luminescence (A and B excitons) peaks induced by strong laser excitation,^31^ defect‐weakening phonon‐assisted indirect emission process,^32^ or interlayer coupling influencing the conduction band minimum (CBM) and valence band maximum (VBM) in such a way as to halt indirect emission line.^33^ Similarly, GaTe flakes have an emission line at 1.96 eV. At the overlapping area, all peaks appear at 1.81, 1.98, and 1.96 eV. The atomic force microscopy measurement is also shown in Figure 2d,e. The uniform contrast observed in the overlapping region demonstrates good contacts between the two flakes and the Si substrate. Figure S1 in the Supporting Information shows the Raman spectroscopic measurements at different positions of the Si substrate. Well‐developed peaks indicate that the MoS_2_ and GaTe flakes are well‐maintained on the Si surface after the transfer process. The corresponding spatially resolved Raman maps of GaTe (at Raman shift of 145 cm^−1^) and MoS_2_ (at Raman shift of 408 cm^−1^) are shown in Figure 2g,h. Additionally, the cross‐sectional TEM image and EDS mapping of the as‐fabricated vertical bipolar junction transistor are shown in Figure 2f,i, respectively.

**Figure 1:**
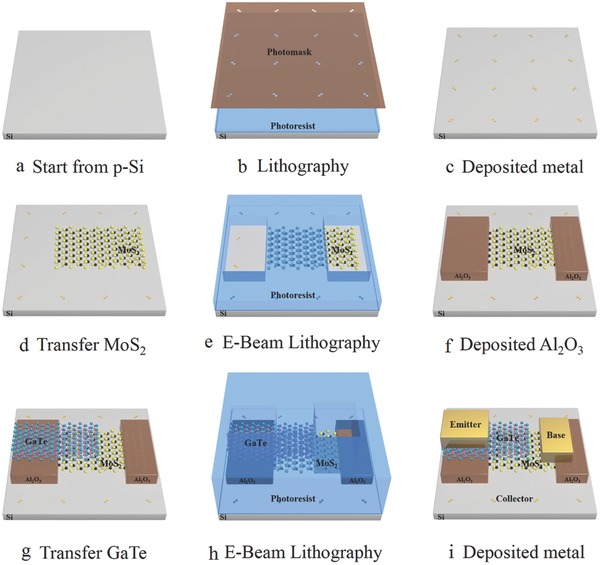
Schematic diagram showing fabrication process flow of the vertical bipolar junction transistor.

**Figure 2:**
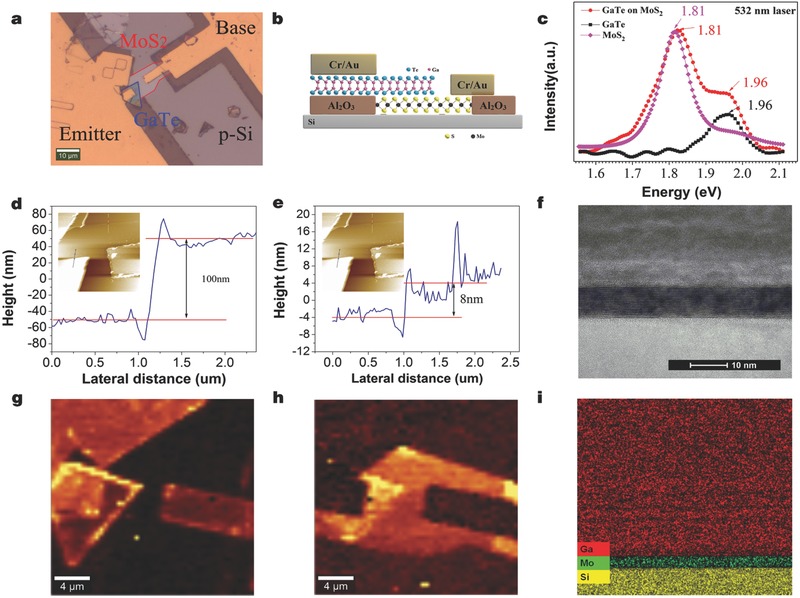
a) The optical microscopy of the bipolar junction transistor device showing the base and emitter contacts. Scale bar: 10 µm. b) Schematic diagram of the structure for the bipolar junction transistor. c) Typical photoluminescence spectra of an individual MoS_2_, an individual GaTe and a MoS_2_/GaTe heterostructure. d,e) Height profiles of the device. A step height of GaTe ≈100 nm and MoS_2_ ≈8 nm is measured. Inset: AFM image of the device. g,h) Spatially resolved Raman maps for the GaTe (Raman shift at 145 cm^−1^) and the MoS_2_ (Raman shift at 408 cm^−1^). f) Cross‐sectional TEM images of the device. Scale bar is 2 nm. i) EDS mapping of the device.

With the well‐defined p‐type characteristics of GaTe and n‐type characteristics of MoS_2_ (Figure S2, Supporting Information), the device exhibits typical characteristics of a p–n–p HBT. Prior to investigating the electrical characteristics of the HBT, electrical transport properties of p‐Si/MoS_2_ and GaTe/MoS_2_ heterostructures were characterized to ensure the p–n diode was achieved from the start. The electrical characterizations of the heterostructures were performed via a two‐terminal configuration, using the base–emitter or base–collector contacts, as shown in **Figure** **3**. Typical current–voltage (*I*–*V*) characteristics, under varying voltage, of p‐Si/MoS_2_ heterostructure is shown in Figure 3a. Clear current rectification behavior was observed in the *I*–*V* plots of p‐Si/MoS_2_ heterostructures. This suggests that the current can pass through the device only when the p‐type Si is positively biased. The observations of the current rectification demonstrated a p–n diode within the p‐Si/MoS_2_ vertical heterostructures. When *V* was in range of ±1 V, the p‐Si/MoS_2_ heterostructures showed a rectification ratio of over 700. The *I*–*V* characteristics of the vertical GaTe/MoS_2_ heterostructures exhibited typical rectifying behavior with an on‐off current ratio of ≈1800 when *V* was within the ±1 V range (Figure 3b). Under a positive bias voltage, the built‐in potential at the interface between GaTe and MoS_2_ was reduced. The electrons could then be transported across the layers easily, resulting in a large on‐state current. Similarly, under a negative bias voltage, the built‐in potential was also much greater and resulted in a small off‐state current. Importantly, the ideality factor *n* has also been derived via the semilogarithm *I*–*V* plot data. By using the slope, the following equation (Calculation details described in Section 3 of the Supporting Information) determines the ideality factor *n*

(1) $$n\;=\frac{q}{kT\times \mathrm{slope}}$$

where *q* is the elementary charge, *k* is the Boltzmann constant, and *T* is the temperature. The ideality factor of p‐Si/MoS_2_ heterostructures device is calculated as 1.178. However, the value of *n* obtained in MoS_2_/GaTe heterostructures device is 1.62. The deviation from the ideal diode behavior (n = 1) can be attributed to the additional series resistance in the device in the high forward bias region;^34^ it can also result from the presence of interfacial defects at the junction.^35^

**Figure 3:**
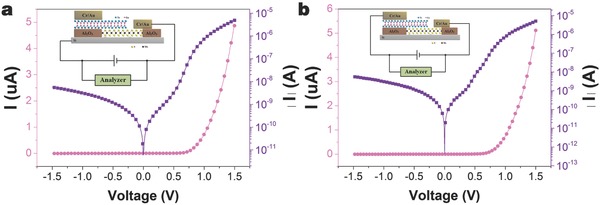
Current versus bias voltage characteristic of a) MoS_2_/p‐Si junction and b) GaTe/MoS_2_ junction. The insets show the measurement schematic diagrams for p‐silicon/MoS_2_ and GaTe/MoS_2_ junction, respectively.

For the fabricated p–n–p transistor with a common‐base configuration, a set of output characteristics are shown in **Figure** **4**a. The output characteristic curves indicate the variation in the collector current (*I*_C_) with a changing collector–base voltage (*V*_CB_) when the emitter current (*I*_E_) was kept constant. *I*_E_ changed from 0 to 14 µA with a step size of 2 µA, and the collector voltage was swept from −0.8 to 2 V. In the active region, where the collector–base junction was reverse‐biased, the curves were almost flat, which indicates that the collector's current *I*_C_ is approximately equal to the emitter's current *I*_E_. As *V*_CB_ became positive, the collector–base junction was biased by a positive voltage. Hence, the collector current *I*_C_ (for a given *I*_E_) sharply decreased. In the saturation region, the collector current did not depend on the emitter's current. However, when *I*_E_ = 0, the collector current was not zero but was very small, which is the reverse leakage current *I*_CO_. The current transmission coefficient (α) of the BJT is defined as

(2) $$\alpha \;=\frac{I_{C}-I_{C\left(I_{E}=\;0\right)}}{I_{E}}$$

**Figure 4:**
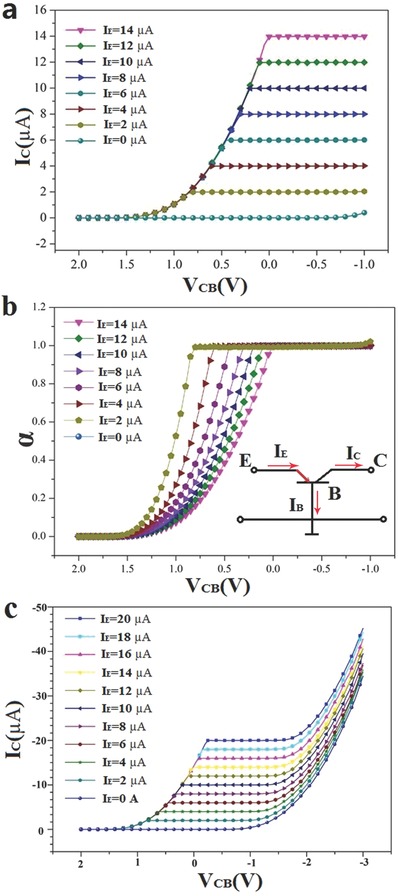
a) Measured forward common‐base output characteristics (*I*_C_ *‐V*_CB_) with a step size *I*_E_ from 0 µA to 14 µA. b) The common‐base current gain (α) versus base–collector voltage (*V*_CB_) curves at room temperature. c). Base–collector junction is punctured through when further increasing *V*_CB_ after saturation region.

It presents the efficiency of electrons from the emitter transferred to the collector in common‐base configure. The plot of the common‐base current gain (α) versus the base–collector voltage (*V*_CB_) displays a curve at room temperature and is shown in Figure 4b. α can reach 0.95, which means that *I*_C_ is approximately equal to *I*_E_, with an extremely small difference at the saturation region. The ratio of holes, which are recombined by electrons in the base region, is small compared to all holes injected to the base from the emitter. The high‐efficiency of the current transmission is the best of any recently reported BJTs or hot electron transistors (HETs) based on 2D semiconductors.^36^, ^37^, ^38^, ^39^ In the active region, the emitter–base junction is forward‐biased, resulting in the lowering of the potential barrier and the narrowing of the space‐charge region (at the emitter–base junction) so that a significant number of holes can be injected from the emitter into the base region. The holes are gathered in the MoS_2_ layer and are rapidly diffused through the base terminal, thus *I*_B_ is approximately equal to *I*_E_. With the further reduction of *V*_CB_, the base–collector junction is punctured and the *I*_C_ increases rapidly, as shown in Figure 4c.

Different from the common‐base configuration, the emitter made the input and output common. The signal was applied between the base and the emitter, and the output was developed between the collector and emitter in the common‐emitter (CE) configuration, as shown in the inset of **Figure** **5**b. The output characteristic curves (Figure 5a) show the variation of the collector current, with a changing collector–emitter voltage *V*_CE_ and a constant base current. In the active region, the curves exhibit an ascendant trend, where *I*_C_ increases with *V*_CE_. In the saturation region, *V*_CE_ became small and the collector–base junction was biased with a positive voltage, resulting in a sharp decrease of the *I*_C_. In this region, the collector current did not highly depend on the base current. However, the common‐emitter direct current gain of the BJT is defined as

(3) $$\beta_{\mathrm{DC}}=\frac{I_{C}}{I_{B}}$$

**Figure 5:**
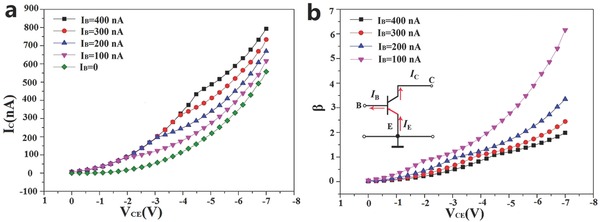
a) Measured forward common‐emitter output characteristics (*I*_C_ *‐V*_CE_) with a step size *I*_B_ from 0 to 400 nA. b) The common‐emitter current gain (β) versus collector–emitter voltage (*V*_CE_) curves at room temperature.

Thus, β is the ratio of DC collector current to DC base current. The plot of the common‐emitter current gain (β) versus the emitter–collector voltage (*V*_CE_) curve at room temperature is shown in Figure 5b. The maximum β (≈7) can be achieved when *V*_CE_ = −7 V and *I*_B_ = 100 nA. The current gain of ≈7 is not the maximum value our device can achieve. As in the common‐emitter configuration, *I*_C_ is not saturated, even when *V*_CE_ was applied at −7 V. For the protection of our device, a higher voltage was not applied. Additionally, other NPN HBT‐based MoS_2_/GaTe/n‐Si heterostructures were also fabricated using the same methods provided in Section 4 of the Supporting Information, whose characteristics are not as good as ours but also shows conventional NPN BJT characteristics. Our device performance and properties are compared with other reported HBT/HET devices based on 2D materials and listed in **Table** **1**.^36^, ^37^, ^38^, ^39^, ^40^, ^41^ The theory of HETs is different than the theory of BJT/HETs, and there is a barrier between the emitter and the base layer in HETs. Nevertheless, the basic mechanism and performance index are similar. The performance of the current density, common‐emitter current gain, and current transmission coefficient is almost the best. This result suggests that our device is very competitive (in terms of performance) in the HBT/HET that is based on 2D materials.

**Table 1:** Comparison of HBT/HET device performance and properties between this work and other reported devices based on 2D materials

	Emitter	base	*J*_C_ [cm^−2^]	β	α
UCLA^36^	Si/SiO_2_	MoS_2_	≈1 µA	4	0.95
KTH^37^	Si/SiO_2_	Graphene	≈10 µA	0.065	0.065
UCLA^38^	Si/TmSiO/TiO_2_	Graphene	≈50 µA	≈0.78	0.44
KTH^39^	GaN/AlN	Graphene	4 A	0.4	≈0.28
MIT^40^	GaN/AlN/GaN	Graphene	≈50 A	4–6	0.75
NCKU^41^	MoS_2_	WSe_2_	0.004	2	
This work	GaTe	MoS_2_	70 A	7	0.95

To better interpret *I–V* characteristics of the HBT, band diagrams were obtained along the vertical dashed line and were used to reveal its working mechanism. This is show in Figure 2b. Based on the previous work,^42^, ^43^, ^44^ discussing the band diagrams of GaTe/MoS_2_ junctions, the band diagram of our device (without bias) is shown in **Figure** **6**a. In common‐base configurations (Figure 4a), *V*_CB_ < 0 (*V*_BC_ > 0, collector is biased with a positive voltage) and *V*_BE_ > 0 (the emitter is biased with a positive voltage), which is the saturated state (band diagram as shown in Figure 6b). In this case, a reverse bias is applied on the base–collector junction. This results in the potential barrier increasing and the space‐charge region broadening at the base–emitter junction so that the holes can be injected from the base into the collector region. In the common‐emitter configuration (Figure 4c), if the *V*_BE_ > 0 and *V*_BC_ < 0, then the *V*_CE_ >*V*_BE_ > 0. In the amplification state, a forward bias is applied to the base–emitter junction (*V*_BE_ > 0), resulting in the potential barrier lowering. The space‐charge region's narrowing at the base–emitter junction is made so that a significant number of holes can be injected from the emitter into the base region, as shown in Figure 6c. As the base–collector junction is reverse‐biased (*V*_BC_ < 0), the holes diffusing to the edge of the depletion region of the base–collector junction are transferred to the collector immediately. A small number of holes are recombined by the electrons in the MoS_2_ flake for a few MoS_2_ layers. Therefore, the current input to the base is amplified, as are the common‐emitter output characteristics shown in Figure 5a,b, respectively. The difference between the saturation state and the amplification state is that the base–collector junction is biased by a positive voltage, hence the barrier potential is reduced and the space‐charge region at the junction also becomes narrow.

**Figure 6:**
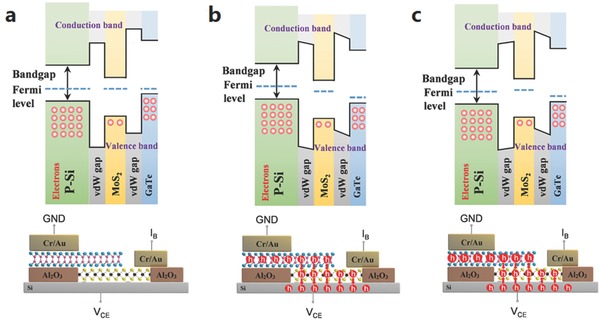
Band diagrams along the vertical dashed line in Figure 2b are shown in original state a), common‐base configuration (amplification state) b), common‐emitter configuration (saturation state) c), respectively.

In conclusion, we have successfully fabricated a bipolar junction transistor by combining GaTe, MoS_2_, and Si. Different from the BJT based on conventional bulk semiconductors, this device uses 2DLMs for the base and the emitter. This new BJT type has realized the typical characteristics of BJTs and the current amplification. Furthermore, the improved device performance can be achieved by optimizing the fabrication process. The 2D‐3D HBTs integrated with 2DLMs with conventional 3D bulk materials provide new exciting perspectives for the investigation of 2DLMs‐based amplifiers and integrated circuits.

## Experimental Section

*Device Fabrication and Electrical Characterization*: This vertical HBT started with a commercially available Si‐wafer, slightly p‐doped with phosphorus. Before transferring MoS_2_, native oxide on the exposed Si substrate was carefully removed via the additional wet etching. Then, a few layers of MoS_2_ were exfoliated from the commercially available crystals on p‐type silicon substrates. After placing the MoS_2_ on the substrate, the wafer was annealed under vacuum (10^−1^ Pa) at 300 °C for 30 min, to remove any residue tapes. The insulating layer used for isolating the emitter and metal electrode from the collector was patterned using e‐beam lithography. Subsequently, without removing the photoresist, ≈40 nm of Al_2_O_3_ was deposited using the atomic layer deposition. Next, the photoresist was removed using acetone, after which only Al_2_O_3_ remained in the trenches and was washed away (with the resist) from the other regions. With the help of optical microscopy, the exfoliated GaTe was transferred directionally to the target MoS_2_ flake. Finally, electrode patterns were defined by a standard electron beam lithography (EBL) process and 10/60 nm Cr/Au electrodes were deposited via physical vapor deposition. Electrical properties of the fabricated devices were measured in a probe station using a semiconductor device parameter analyzer (Agilent, B1500A).

## Conflict of Interest

The authors declare no conflict of interest.

## Supporting information

Supplementary
