# Supplementary material for: High Performance Amplifier Element Realization via MoS2/GaTe Heterostructures
Source: Adv Sci (Weinh). 2018 Jan 15;5(4):1700830. doi: 10.1002/advs.201700830 (PMC5908369; doi:10.1002/advs.201700830)
Supplement: Supplementary file 1 — Supplementary [file ADVS-5-1700830-s001.pdf]

Copyright WILEY-VCH Verlag GmbH & Co. KGaA, 69469 Weinheim, Germany, 2018.

Supporting Information

for *Adv. Sci.,* DOI: 10.1002/advs.201700830

High Performance Amplifier Element Realization via MoS_2/ GaTe Heterostructures

*Xiao Yan, David Wei Zhang, Chunsen Liu, Wenzhong Bao, Shuiyuan Wang, Shijin Ding, Gengfeng Zheng, and Peng Zhou**

Supporting Information

High Performance Amplifier Elements Realization by MoS_2/GaTe Heterostructure

*Xiao Yan[1†], David Wei Zhang[1†], Chunsen Liu[1], Wenzhong Bao[1], Shuiyuan Wang[1], Shijin Ding[1], Gengfeng Zheng[2] and Peng Zhou[1]**

[1] State Key Laboratory of ASIC and System, School of Microelectronics, Fudan University, Shanghai 200433, China

[2] Laboratory of Advanced Materials, Department of Chemistry, Collaborative Innovation Center of Chemistry for Energy Materials, Fudan University, Shanghai, China

† These authors contribute equally to this work.

*Correspondence and requests for materials should be addressed to P. Z. (E-mail: pengzhou@fudan.edu.cn)

1. Raman spectroscopy

Figure S1 is the Raman spectroscopic measurement of different positions on the Si substrate. Figure S1a shows that the first order peaks E_{2g}^{1} and A_{1g} correspond to in-plane and out-of-plane vibrations. In this case, these vibrations are located at 383.9 cm^{-1} and 408.3 cm^{-1} with a separation of 24.31 cm^{-1}. The Raman spectrum from individual GaTe has two peaks at 120.6 and 139.8 cm^{-1}, in agreement with the literature. The respective Raman peaks suggest that the representative vibration modes of both GaTe and MoS_2 indicates the coexistence of two distinct materials within the heterostructure.

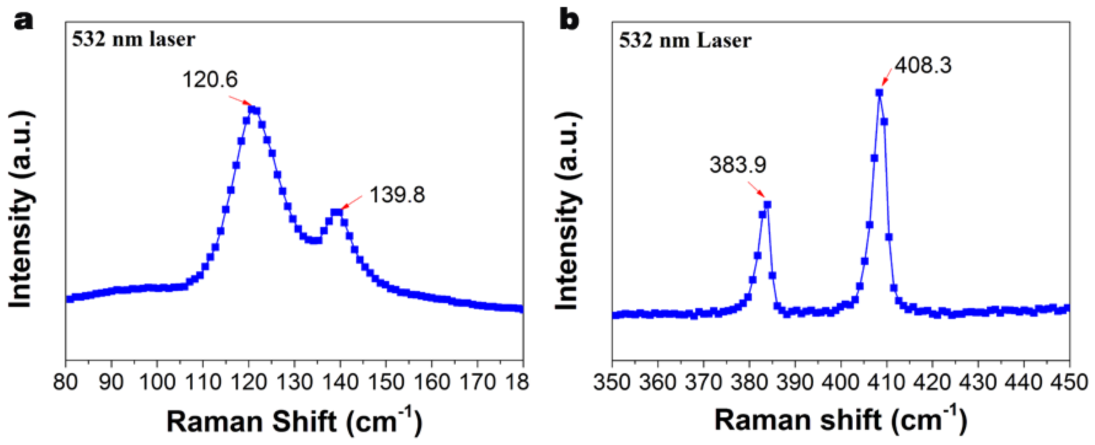

Figure S1. Raman spectroscopy (using a 532 nm laser) of a particular device in different positions, (**a**) GaTe and (**b**) MoS_2.

2. Electrical characterization of the FET based on MoS_2 and GaTe

To investigate the transport characteristics of the exfoliated MoS_2 and GaTe, the transistors with the back-gate field effect, based on the exfoliated MoS_2 and GaTe, have been fabricated. The transfer characteristics (I_d-V_g) of the FET are shown in **Figure S2**. The field-effect mobility (μ) was then extracted using equation $\mu = \frac{dI_d}{dV_g}\frac{L}{WC_gV_{ds}}$, where C_g is the top gate capacitance per unit area, L is the channel length, W is the channel width, V_{ds} is the source-drain voltage and $\frac{dI_d}{dV_G}$ is the transconductance (i.e., the slope of transfer curve in a linear region). The electron mobility of the GaTe FET shown in **Figure S2a** is estimated to be only 0.01 $cm^2/V\cdot s$, while that of MoS_2 FET reaches 5.2067 $cm^2/V\cdot s$ in **Figure S2b**.

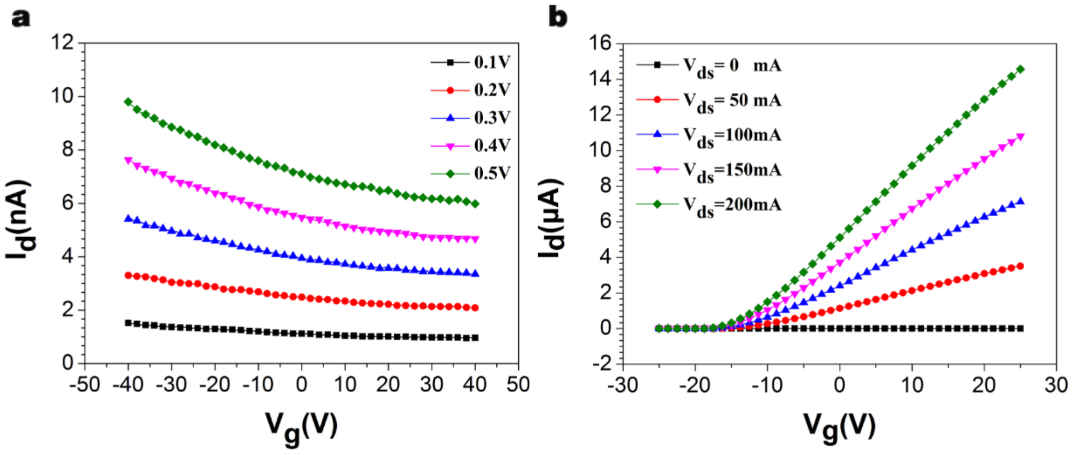

Figure S2. Electrical characterization of the back-gate FETs based on (**a**) GaTe and (**b**) MoS_2.

3. Calculation details

The *I–V* characteristics have been fitted with the diode equation,

$$I = I_0\left(e^{\frac{q(V-V_T)}{nkT}} - 1\right). \quad \text{(S1)}$$

When V>>V_T

$$\ln\frac{I}{I_0} = \frac{q(V-V_T)}{nkT}. \quad \text{(S2)}$$

To calculate the ideality factor *n*,

$$\mathrm{n} = \frac{q}{kT\left(\frac{lnI - lnI_0}{V - V_T}\right)}. \quad \text{(S3)}$$

For slope $= \frac{lnI-lnI_0}{V-V_T}$, so

$$\mathrm{n} = \frac{q}{kT \times slope} \quad (1)$$

As shown in **Figure S3a**, V_T is 0.75 V, and through curve fitting, n=1.654, and

$$\mathrm{I} = 0.02437 * \left(e^{\frac{V-0.75}{0.038}} - 1\right)$$

When V_T is 1.1 V in **Figure S3b**, n=1.178. For MoS_2/GaTe junction, as shown in **Figure S3c**, V_T is 0.75 V, and through curve fitting, n=1.117. When V_T is 1.1 V in **Figure S3d**, n=1.62.

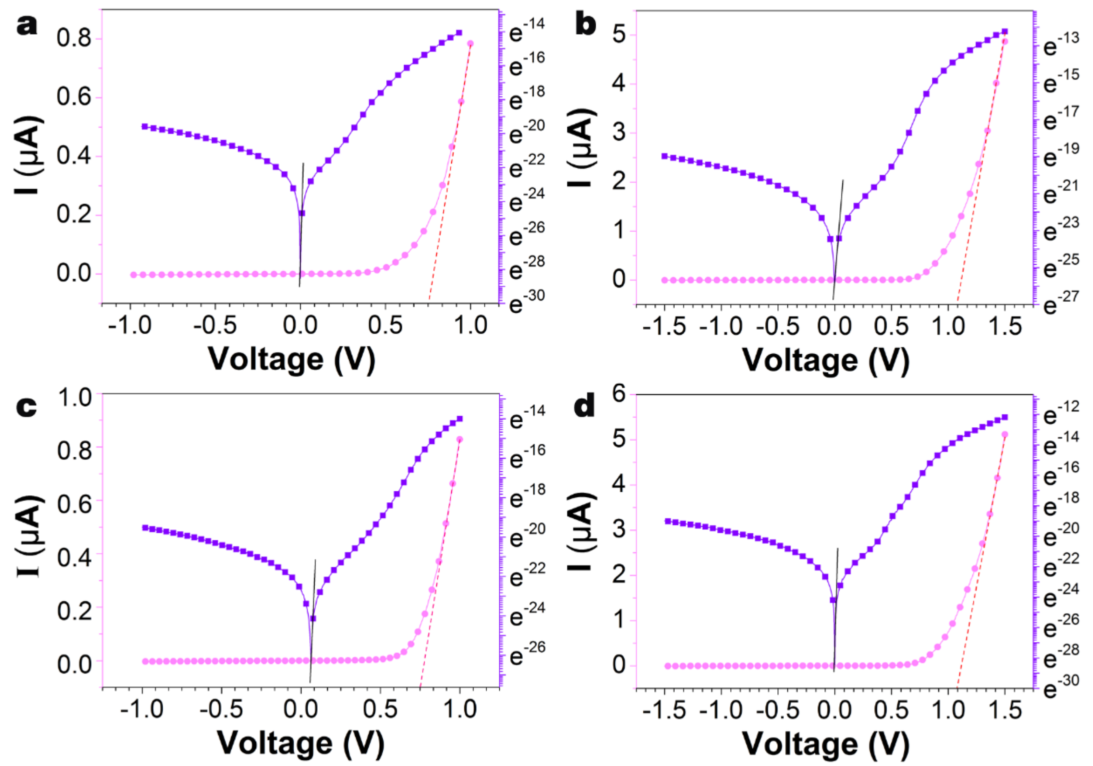

Figure S3. a,b Current-bias curves of a p-Si/MoS_2 (a) V=-1 V to 1 V, (b) V=-1.5 V to 1.5 V in natural-logarithmic coordinates. c,d Current-bias curves of a GaTe/MoS_2 in natural-logarithmic coordinates, (c) V=-1 V to 1 V, (d) V=-1.5 V to 1.5 V.

4. NPN HBT based on MoS_2/GaTe/n-Si heterostructure

After removing the native oxide on the exposed Si substrate via additional wet etching, few layers of the GaTe were exfoliated on the n-type silicon substrate, as shown in **Figure S4a**. After placing the GaTe on the substrate, the insulating layer used for isolating the emitter and metal electrode from the collector was patterned using e-beam lithography, as shown in **Figure S4b**. Subsequently, without removing the photoresistance, approximately

40 nm of Al_2O_3 was grown via atomic layer deposition. Next, the photoresist was removed using acetone so that only Al_2O_3 remained in the trenches and was washed away (with the photoresist) from the other regions, as shown in **Figure S4c**. With the help of OM, MoS_2 was transferred directionally to the target GaTe flake, as shown in **Figure S4d**. Finally, electrode patterns were defined by a standard EBL process, as shown in **Figure S4e**. Additionally, the 10/60 nm Cr/Au electrodes were deposited via physical vapor deposition, as shown in **Figure S4f**.

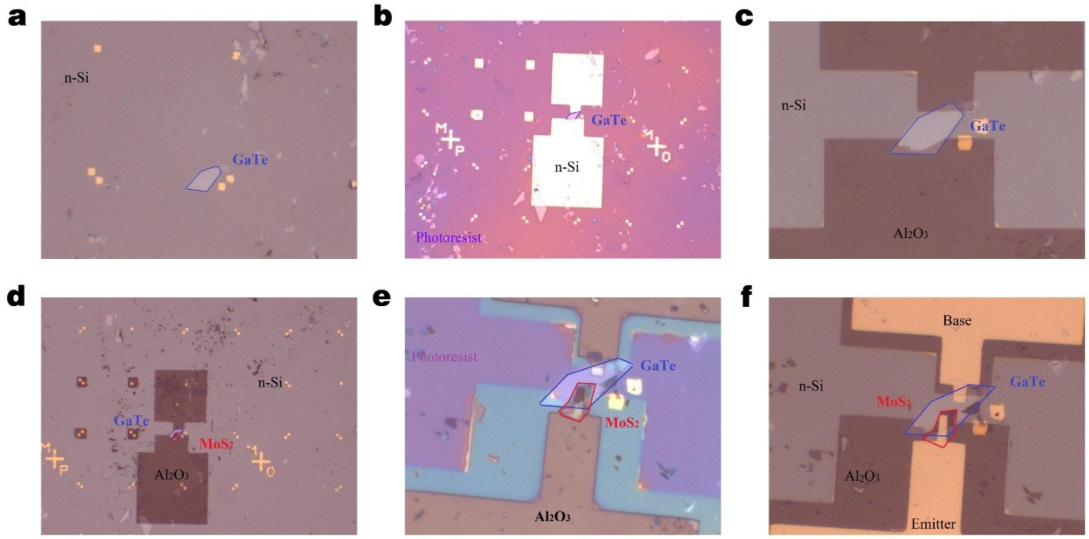

Figure S4. Optical micrograph of the fabrication process of the vertical HBT. The red and blue circled regions represent the MoS_2 and GaTe flakes, respectively.

To verify this concept, that two-dimensional materials could be used to fabricate the amplifying device, we fabricated a new NPN BTJ by sequentially transferring

mechanically exfoliated GaTe and MoS_2 thin films onto a slightly n-doped Si substrate. The GaTe was located on the gap between MoS_2 and Si, and the schematic of the MoS_2/GaTe/n-Si heterostructure is shown in **Figure S5**.

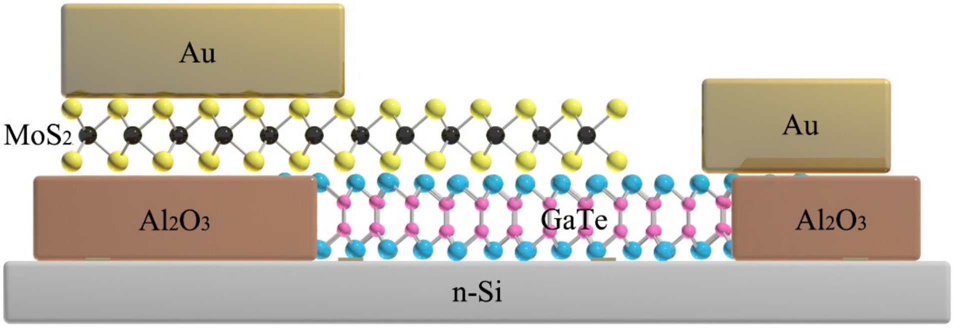

Figure S5. Schematic of the NPN HBT based on MoS_2/GaTe/n-Si heterostructure

As shown in **Figure S6a**, a different I_E from 0 nA to 400 nA with a 100-nA step size are applied and the V_{CB} is swept from 0 V to 3 V. In the active region, where the collector-base junction is reverse-biased, I_C is approximately equal to the emitter current I_E. As V_{CB} decreases, the collector-base junction becomes more forward biased. Hence, the I_C sharply decreases. In the saturation region, the collector current slightly depends on the emitter current. The plot of common-base current gain (α) vs. base-collector voltage (V_{CB}) curves under room temperature is shown in **Figure S6b**. As shown in **Figure S6c**, in the active region, the curves exhibit an ascendant trend under the common-emitter configuration, which indicates that the I_C increases as the V_{CE} increases. In the saturation

region, the V_{CE} becomes small, and the collector-base junction becomes forward biased. Hence, the I_C decreases sharply. In this region, the collector current depends on the base current. The plot of the common-emitter current gain (β) vs. emitter-collector voltage (V_{CE}) under room temperature results in a curve that is shown in **Figure S6d**, and the maximum β increases from 0 to 1.4.

The common-emitter current gain (β) of the P-N-P HBT can reach 7 at room temperature, but that of the N-P-N HBT can be only 1.2. The base of the P-N-P HBT is MoS_2 and the emitter is GaTe, while the base of the N-P-N HBT is GaTe and MoS_2 is emitter. The thickness of GaTe flake in the N-P-N HBT is much thicker than the MoS_2 flakes in the P-N-P HBT, which means that the base layer of the N-P-N HBT is much thicker than that of P-N-P HBT. However, the base layer has to be thinner so that fewer carriers (injected from the emitter) will be recombined with the carriers of opposite polarity in the base layer so that the current amplification gain will be enhanced. GaTe, whose impurity concentration is relatively high for the base material, and the electrons transferred from the emitter region will compound with the majority carrier holes in the base region under the amplification condition. Hence, electrons collected in the collector region will decrease greatly. Therefore, the current amplification gain of N-P-N HBT with a thick GaTe base layer will be much lower than the P-N-P HBT with a thin MoS2 base layer. Current gain β is not particularly large in the N-P-N HBT, but β is greater than

1, so there are still some amplification effects. In the future, we will choose another kind of p-type 2D material as the base layer in the N-P-N HBT to achieve higher performance.

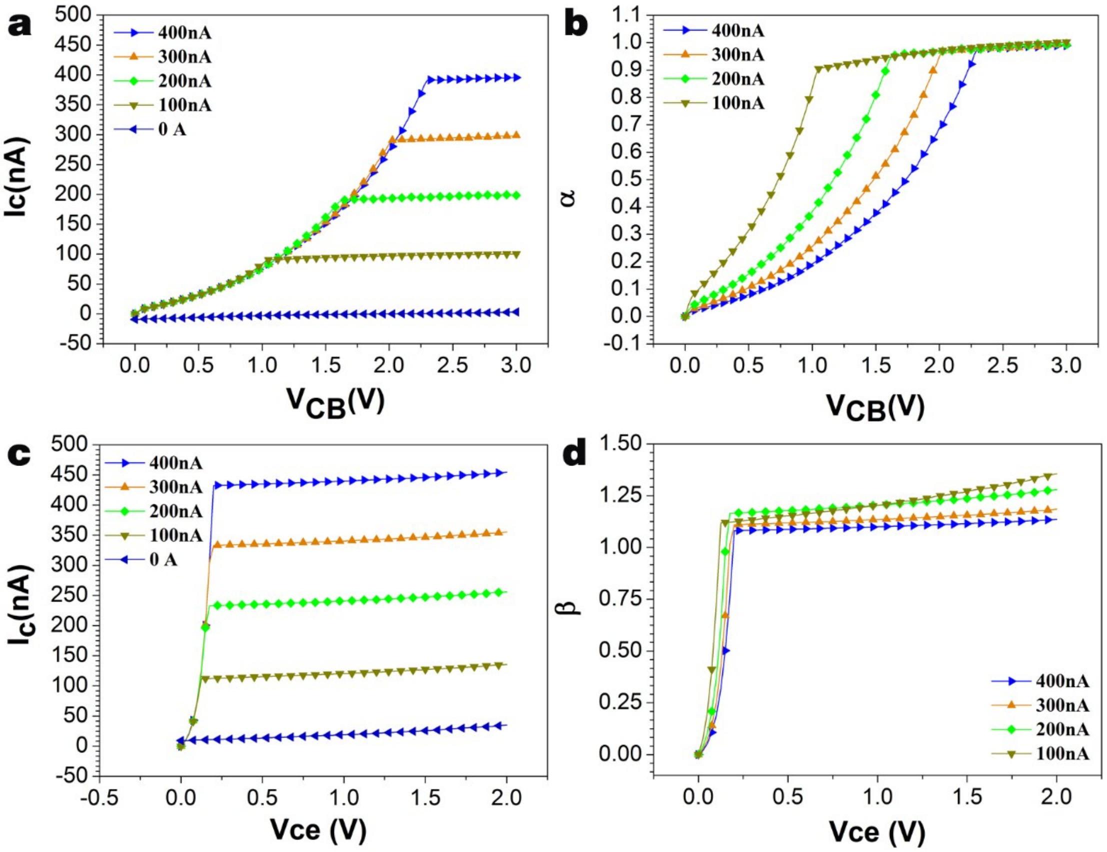

Figure S6. (a) Measured forward common-base output characteristics (I_C-V_{CB}) with I_E from 0 nA to 400 nA with a step size of 100 nA at room temperature. (b) The common-base current gain (α) vs. the base-collector voltage (V_{CB}) curve under room temperature. (c) Measured forward common-emitter output characteristics (I_C-V_{CE}) with I_B from 0 to 400 nA with a step size of 100 nA at room temperature. (d) The common-emitter current gain (β) vs. collector-emitter voltage (V_{CE}) curve under room temperature.
